# Why Do Users of Online Mental Health Communities Get Likes and Reposts: A Combination of Text Mining and Empirical Analysis

**DOI:** 10.3390/healthcare9091133

**Published:** 2021-08-31

**Authors:** Jingfang Liu, Jun Kong

**Affiliations:** School of Management, Shanghai University, Shanghai 201800, China; jingfangliu2014@hotmail.com

**Keywords:** online health community, information adoption, mental health, text mining, empirical analysis

## Abstract

An online community is one of the important ways for people with mental disorders to receive assistance and obtain support. This study aims to help users with mental disorders to obtain more support and communication through online communities, and to provide community managers with the possible influence mechanisms based on the information adoption model. We obtained a total of 49,047 posts of an online mental health communities in China, over a 40-day period. Then we used a combination of text mining and empirical analysis. Topic and sentiment analysis were used to derive the key variables—the topic of posts that the users care about most, and the emotion scores contained in posts. We then constructed a theoretical model based on the information adoption model. As core independent variables of information quality, on online mental health communities, the topic of social experience in posts (0.368 ***), the topic of emotional expression (0.353 ***), and the sentiment contained in the text (0.002 *) all had significant positive relationships with the number of likes and reposts. This study found that the users of online mental health communities are more attentive to the topics of social experience and emotional expressions, while they also care about the non-linguistic information. This study highlights the importance of helping community users to post on community-related topics, and gives administrators possible ways to help users gain the communication and support they need.

## 1. Introduction

Mental disorders represent a serious threat to human health due to their high incidence, long duration, and tendency to relapse after cure. Depression, a typical mental disorder, is the leading cause of disability worldwide [1,2,3]. According to the World Health Organization (WHO), 350 million people worldwide were suffering from depression in 2018, and severe depression can even lead to suicidal behavior [4,5,6]. Optimistically, with the ever-increasing accessibility of web-based technology, online communities offer a convenient and promising solution for individuals suffering from depression. Studies have shown that with the help of science and technology, such as the Internet, individuals with early- and mid-stage depression may be able to manage their condition if they receive timely treatment, such as participation in online health treatment [7,8]. For patients with mild to moderate mental disorders, access to medical assistance via the internet can help them to avoid long wait times for medical consultation and reduce the personal stigma associated with social pressure; thus, online communities allow individuals with depression to receive timely and convenient assistance [9,10,11].

In online communities, one of the main forms of communication between users is via posts and replies, through which they provide each other with emotional, informational, and other forms of support [12,13,14]. For individuals with mental disorders, timely and long-term support and communication will not only help them to achieve better medical outcomes during treatment but will reduce the probability of recurrence after recovery [15,16,17]. In addition, researchers have found that a lack of necessary support and communication with others may increase an individual’s chances of developing long-term depression and even increase the risk of suicide [18,19]. Support from family or friends can help to alleviate negative feelings or stigma associated with depression, and the quality of social support received by individuals with depression has been proven to be strongly associated with improvements in mood [20,21,22]. Since online communities are an important source of support and communication for those with depression, this study focuses on how the characteristics of patients’ online posts affect the number of likes and reposts that they receive.

There are two main gaps in the current research: (1) The research on online community users’ posting mostly uses questionnaires to investigate their posting motivation and posting benefits [23,24], considers user posting and replying characteristics from a social network perspective, or analyzes the posting characteristics of users in the community solely from the textual characteristics of the posts [25,26]. Few studies have combined the textual characteristics of posts with user characteristics and further considered the relationship with communication quantity. (2) Due to the characteristics of mental disorders, in addition to personal medical help and social support, online health communities are also an important means for patients to access treatment resources [7,9,15]. Moreover, based on our previous research, the posts of online mental health community users are different from those of other online health communities; users of online mental health communities more often discuss their feelings and living conditions, unlike the physiological illness community, which focuses more on treatment options [27]. While existing research rarely incorporates the characteristics of patients with mental disorders into research models, relevant information system theories lack validation in mental online health communities. This paper will fill this gap by qualitatively studying the content of posts in an online mental health community through text mining, and incorporating the characteristics of users and posts into a regression model to quantitatively study the factors that influence the quantity of support that they receive online. The aim is to explore the factors influencing the number of likes and reposts for posts in online communities for mental illness and to enhance the ability of users with mental illness to obtain support and communication through online communities.

The study is structured as follows: Section 1 explains the background and significance of the study; Section 2 reviews the information adoption model and based on that presents the questions and hypotheses; Section 3 is the methodology of the study; Section 4 details the results of the regression model; Section 5 discusses the findings and limitations of this paper; the last section concludes this paper.

## 2. Theoretical Background and Hypothesis

This paper attempts to identify factors influencing the number of likes and reposts in an online community for depression. The research model is derived from the information adoption model, addresses two main attributes of posts, and empirically examines their impact on the number of likes and reposts.

### 2.1. Information Adoption Model

In the information systems literature, researchers have applied the technology acceptance model (TAM) to define how people are influenced when they are exposed to ideas or information [28]. Based on this, the information adoption model (IAM) developed by Sussman and Siegal (2003) is also widely used in the information systems domain to explain how people deal with the information that they receive [29,30]. According to the information adoption model, the central and peripheral cues of a message influence the recipient’s adoption behavior through information usefulness, that is, information quality and source credibility affect the perceived usefulness of the message and, thus, the message adoption behavior. IAM explains how information from online platforms is received by users and is therefore very applicable to online content research [31,32]. The model is also appropriate for this study, as it focuses on an online community and explores the reasons that users adopt information and give support. This study uses three elements from IAM to construct the research model, namely information quality, source credibility, and user-perceived usefulness.

### 2.2. Hypothesis

#### 2.2.1. Perceived Usefulness

Based on IAM, users make judgments about the usefulness of information in conjunction with their personal beliefs, which further influence their information adoption behavior [30,33]. According to previous studies, users will recognize information when it is consistent with their personal beliefs, meaning that they find the information to be useful to themselves [34]. In online communities, this recognition may influence some of the users’ behaviors, such as following someone who provides the information that they need, or liking and reposting a post that they agreed with. Thus, liking or reposting a post is considered to be one of the measures of usefulness or information adoption [35]. Liking involves a user showings support or appreciation for a post via a function button under the post [36,37]; this button is usually represented by a symbol—for example, a heart on Twitter, or a thumbs up on Facebook and Microblog. Reposting a post usually means that the user finds the post is useful, such as a post that contains medical information or a post that expresses feelings that they identify with [38]. Furthermore, because within disease-specific communities users’ information needs and emotional feelings are more likely to be similar, by reposting posts about their experiences, the reposters create a sense of community and mutual support. 

Since users’ decision making in virtual communities is more rapid compared to the real world, and the behavioral time is shorter between perceived usefulness of information and information adoption, the information usefulness can be directly measured by the variable indicating information adoption [39].

#### 2.2.2. Information Quality

Information in online communities can be generated by almost every user in the community, so the quality and credibility of the information is crucial [40,41]. In studies related to information systems, information quality usually includes the concepts of completeness, clarity, comprehensibility, usefulness, and reliability, and different scholars choose existing concepts or add new concepts in their studies [42,43,44]. In the literatures on text analysis, various dimensions of information quality can be measured by some common text features, such as linguistic style, semantic features, affective features, etc. [45,46]. In this paper, we follow these common proxy variables, including text content, text sentiment score, text length, and image features [44,45,46]. Therefore, this study will select variables from among these aspects.

Within online health communities, users often seek medical information, attempt to make social connections with others, or express their own opinions about things [47,48,49]. As a medium of information delivery, if the topic of a post is expressed relatively clearly, it will not only make the message more accurate but also provide the reader with a better reading experience [25]. Thus, from the perspective of information quality, a clear post topic is not only a proxy for the information clarity and comprehensibility, but may be an important cue that influences the user’s perceived usefulness. Through research on the disease characteristics of depression, we found that some of the causes of depression come from individuals’ social lives—for example, family and peer pressure or abuse [50,51,52]. Moreover, according to related work, the patients’ social experiences are a common topic of discussion in online health communities [53]. Thus, we believe that social experiences are likely to be an important topic for depression community posts. Therefore, we propose hypothesis 1.

**Hypothesis** **1** **(H1).** *The topic of social experiences in posts in online mental health communities is related to the number of likes and reposts that a post receives.*

Mental illnesses such as depression are typically characterized by cognitive biases and difficulties with emotion regulation [54,55,56]. According to Horner et al., people with depression are characterized by a difficulty in maintaining a positive mood, instead suffering from low mood, so they need a more private and convenient environment to release their emotional stress [57]. We thus believe that online communities provide a platform for users with depression to release their emotions. Similarly, we assume the topic of emotional expression is one of the proxies for information clarity. In addition, texts that express emotions are likely to represent different strengths of emotion semantically; the strength of emotion affects the emotional experience of the reader [58], which can enhance or diminish the comprehensibility of the information. Thus, we believe that emotional strength is also a significant predictor of the perceived usefulness of the posts; we thus propose hypotheses 2 and 3.

**Hypothesis** **2** **(H2).** *The topic of emotional expression in posts in online mental health communities is related to the number of likes and reposts that a post receives*.

**Hypothesis** **3** **(H3).** *The emotional strength of posts in online mental health communities is related to the number of likes and reposts that a post receives*.

Writing style can greatly affect the reader’s experience and, thus, their agreement with the information [25,26]. Research has shown that the length of a post, as a specific representation of the writing style, reflects the amount of information that the text carries [58,59]. As the completeness of the message can be measured by the number of words, we suggest that post length also influences the perceived usefulness of posts in depression communities. We propose hypotheses 4.

**Hypothesis** **4** **(H4).** *The length of text posts in online mental health communities is related to the number of likes and reposts that a post receives*.

In general, images can enhance the reader’s understanding of the text or strengthen the emotions expressed in the text. In addition, images can compensate for the lack of sensory experience brought to users by the virtual environment of the web and influence decision making by affecting the emotional response of the reader [60,61,62]. Therefore, the image of the post can help measure the comprehensibility of the information. Thus, we propose hypotheses 5.

**Hypothesis** **5** **(H5).** *Images included in posts in online mental health communities are related to the number of likes and reposts that a post receives*.

#### 2.2.3. Source Credibility

In online communities, users communicate via the same platform, so information source credibility is derived from the trustworthiness of individual users. In addition, users rely on the source’s credibility to make decisions about further communication [63,64]. Previous research has demonstrated that source credibility is an important antecedent variable in information adoption, including the identity of the information provider and their influence within the virtual community [30,43]. A user’s identity is in fact a type of title, and the authenticity of their identity is enhanced when it comes from a more authoritative organization [35,65]. In this study, the title of a poster is displayed according to whether they are a paid member, expert, or institution, which is mainly derived from the platform. Therefore, when a poster has a title, it enhances the authenticity of their identity. In terms of a user’s influence, simple cues, such as the number of followers, can imply the influence of a user within the community [66,67]. For example, a user’s number of followers can give an indication of how many people will see the message posted by them. Thus, we use the number of followers to measure a user’s influence. Therefore, we propose hypothesis 6 and hypothesis 7.

**Hypothesis** **6** **(H6).** *A poster’s identity in online mental health communities is related to the number of likes and reposts that a post receives.*

**Hypothesis** **7** **(H7).** *A poster’s network influence in online mental health communities is related to the number of likes and reposts that a post receives.*

**Figure 1:**
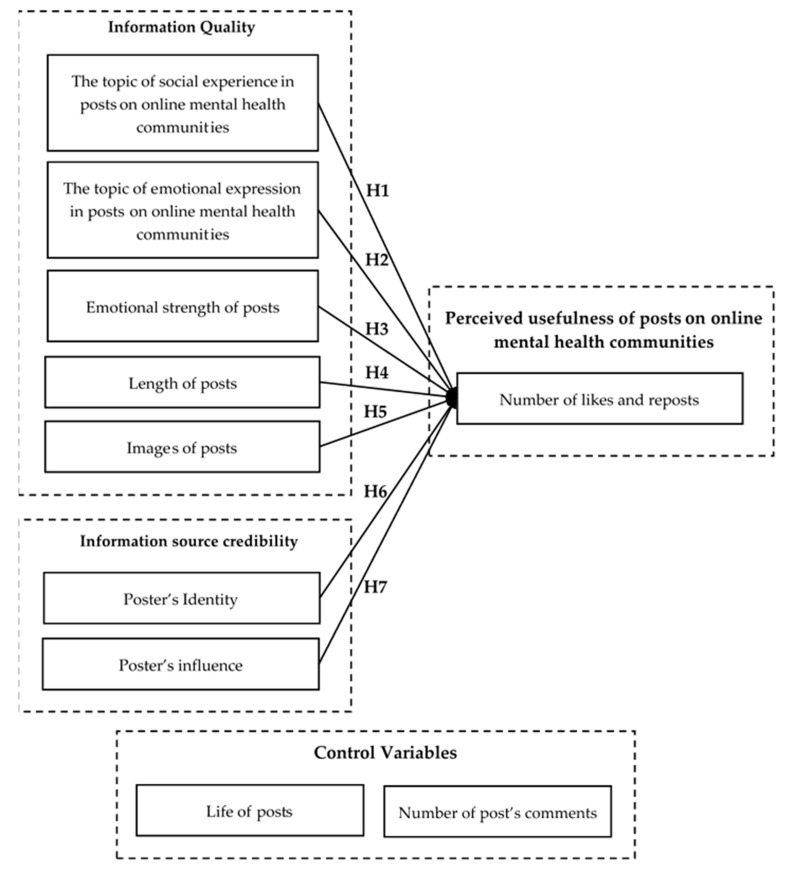
The research model of factors influencing the number of likes and reposts in online mental health communities that a post receives.

In summary, this paper attempts to construct a conceptual model of factors influencing the number of likes and reposts that a post receives in online mental health communities based on the information adoption model, as shown in Figure 1.

## 3. Methods

### 3.1. Data and Variables Measurement

The study text was obtained from depression super-topic community. In this online mental health community, users are not divided according to their real-world identity, providing a degree of anonymity. Moreover, users can ask the community for an identity marker on their own initiative, which is usually used to make the account more attractive; however, regardless of whether a user has an identity marker, all users have the same right to post, reply, or repost freely. We obtained all text data and user data for 49,047 posts in the depression super topic community, which were posted by users using the ‘depression community’ tag and posted within 40 days from 11 March to 21 April 2021. We collected the data from 11 April to 21 April 2021. Due to the site’s rules and our technical shortcomings, we were only able to obtain the full data for one month prior to the current date. In addition, due to the large number of comments on some posts, we only obtained the exact count of comments and the latest 200 comments. Preprocessing was used to remove posts that were missing key information, such as text messages and user information, leaving 47,307 data in total. The definitions of the variables and their descriptions are shown in Table 1.

The dependent variable was the perceived usefulness, which was measured by counting the number of likes and reposts [35,36,37]. The independent variable had two dimensions, which were information quality and source credibility. Information quality includes the topics of social experience and emotional expression, emotional strength, length of posts, and images included in posts. Social experience and emotional expression are topics that people with mental disorders are more concerned with in an online community, as measured by the LDA topic model [25,68]. After implementing the LDA algorithm, each post was assigned with a different probability to each topic generated [27]. Emotional strength was used to measure the intensity of positive or negative emotions in a post [69]. This value was obtained through sentiment analysis, with positive or negative signs representing positive or negative emotions, and the magnitude of the value representing the strength of emotion [11]. Length of posts was calculated as the number of words in a post [25,26,69]. Images in posts was a binary variable that took the value of 1 when the post contained an emoji or an image, and 0 for the rest [35,37,60]. In terms of source credibility, the poster’s identity usually comprised their identified role within the community, which was authorized by the community manager [35]; usually, users are experts in the knowledge domain in Q&A communities, while on social media, they can be paid members or opinion leaders with many followers. In this paper, user identity is a binary value, with 1 for users with identity and 0 for others. A user’s influence is measured by the number of followers, and in social media, the number of followers is a visual indicator of a user’s influence within the platform [66,67]. Since a small number of users have a much larger number of followers than the average, we took the logarithm of the number of followers in order to avoid problems caused by data deviation.

We also included two control variables. First, we assumed that the duration for which a post exists on the platform affects its dissemination range, and the longer it exists, the more users can read it; therefore, it has a greater probability of receiving likes and reposts. We set the lifespan of the post as a control variable, which is the time gap between the posters’ posting date and our collection date; the lifespan of the earliest post was 40 days, and the lifespan of a post posted on the day of collection was 0 days. Second, in addition to likes and reposts, posts also contain additional important data, namely comments on the post from other users, and usually positive comments also reflect the commenter’s agreement or support for the post [38]. However, we found that it was difficult to determine the commenter’s attitude towards the post due to the length and depth of the comments; therefore, it was also difficult for the posters to judge the intention of the commenters (whether they were expressing encouragement or not). However, the number of comments may have some relationship with the number of likes and reposts, so we do not discuss the content of the comments in this study, and we set the number of comments as a control variable.

### 3.2. Topic Analysis and Sentiment Analysis

Latent Dirichlet Allocation (LDA) topic analysis is a three-layer Bayesian topic generation model, a text topic mining algorithm often used in web communities [25,68]. As the LDA model is an unsupervised machine learning method, there is no need to manually annotate the data, and topic clustering can be performed quickly and efficiently when given the number of topics and the set of documents. Moreover, after performing the clustering, each topic is described by a series of keywords, allowing each topic to be interpreted to some degree. First, LDA needs to specify the number of topics, which is often a challenge for researchers. Following the practices of Hao et al., we tested 2–15 topics to perform topic division [70]. With different numbers of topics specified, we analyzed the interpretability of each topic after applying the LDA algorithm. We found that the topic interpretability was the worst when the number of topics was 2. As the number of topics increased, the topics became easier to understand, but when there were more than 10 topics, there were obvious repetitions of topic words, which also meant that these topics might have had some similarity with the previous ones and could not be well interpreted. Therefore, combining our experience with topic decipherability, 10 topics were selected and further labeled manually. Second, we employed three postgraduates to view the keywords under each topic and to rate each topic accordingly. Scores ranged from 0 to 9, with 0 being not at all and 9 being very sure. Human evaluation was used to determine whether they considered the topic to be a social experience or an emotional expression. They were given the keywords of topics belonging to social experiences, such as parents, work, and family, and the keywords of topics belonging to emotional expression, such as pain, wanting to cry, and upset. After the three researchers made their choices independently, we grouped separately the three highest-scoring topics into the topics of social experience and emotional expression. The remaining four topics were beyond the scope of this study because the scorers reported that these topics were mainly discussing complex drug names or reporting misinformation and did not include patients’ social background experiences or emotional expressions; therefore, the probability of these four topics was not included in the scope of this study. Finally, because the LDA algorithm ultimately generates a vector of probabilities for each article that belongs to each topic, we calculated the social experience topic and emotional expression topic probabilities.

Sentiment analysis is a text analysis method used to interpret the emotional intensity of a text [25,71]. Using sentiment dictionaries is a common and convenient means of performing sentiment analysis, and the applicability of sentiment dictionaries to texts affects the accuracy of sentiment analysis. We used the BosonNLP sentiment dictionary to perform sentiment analysis of online posts; is very applicable to our study since this sentiment dictionary is trained from a large number of users posting data on social media. Through sentiment analysis, we could obtain the sentiment score of each post. A positive number means that the post contains a positive sentiment, while a negative number means that the post contains a negative sentiment, and the larger the absolute value of the sentiment score, the stronger the sentiment contained in the post.

### 3.3. Descriptive Statistics

We adopted Stata 16.0 software (StataCorp LLC, College Station, TX, USA) for empirical analysis.

First, we conducted descriptive statistics on 47,307 data, and Table 2 demonstrates the results of descriptive statistics for the study variables. In terms of information quality, the average probability of the social experience topic in the online mental health community was 39.6%, which was higher than the probability of the emotional expression topic at 26.0%, indicating that people with mental illness prefer to talk about their social lives. The average community emotional score in terms of emotional intensity was −1.737, indicating that users of the online mental health community communicated in a more negative way overall. A user’s number of followers is a measure of their influence; there were very few users with a very large number of followers (the highest was 4,410,720), and the distribution was skewed, so the number of followers was logarithmic.

### 3.4. Correlation Analysis

In order to understand whether there was a strong correlation between the independent variables leading to the problem of multicollinearity in the regression analysis, we conducted a correlation analysis of the independent variables, as shown in Table 3. Overall, the absolute values of the correlation coefficients between all independent variables were less than 0.5, which means that there was no problem of multicollinearity.

### 3.5. Regression Analysis

To analyze the relationship between the credibility of information quality sources with the perceived usefulness of posts, we applied Formula (1) as follows:

**NLR** = α + β_1_ × ST + β_2_ × ET + β_3_ × ES + β_4_ × length + β_5_ × Image + β_6_ × identity + β_7_ × Influence + β_8_ × Life + β_9_ × Comments + ε (1)

The dependent variable was the number of likes and reposts, which can be interpreted in online communities as the number of positive responses and support that the post receives. In this study, the number of likes and reposts for the dependent variable was a non-negative integer and the variance was much larger than the mean based on descriptive statistical analysis; therefore, the linear regression model was not applicable to this research question. Since the dependent variable was count data and was overly discrete, a negative binomial regression model was more appropriate for this study [39,72].

## 4. Results

Table 4 shows the path coefficient values, standard error, *z*-values, *p*-values, and significance calculated by the model. The model was examined using the chi-square test, and the independent variable was examined using the *z* test [73].

According to the results, the topics of social experiences and emotional expression among those with depression, emotional strength, post length, inclusion of images, poster identity, and poster influence each had a significant positive relationship with the number of likes and reposts. All the research hypotheses were valid, but the significance levels varied. In particular, the topics of social experience and emotional among those with depression, which we proposed in our study, had effect coefficients of 0.368 and 0.353, respectively.

## 5. Discussion

### 5.1. Principal Findings

This study combines text mining and empirical analysis to explore the factors influencing the number of likes and reposts for posts in online mental health communities and contributes to research on user communication in online communities.

In terms of information quality, we innovatively applied a text analysis approach to propose three variables: the topic of social experience, the topic of emotional expression, and the sentiment score of posts. Those variable all had significant positive relationships with the number of likes and reposts that a post receives in online mental health communities. In addition, the length of the post and whether the post contains images are also predictors of a post’s likes and reposts.

In terms of information source credibility, both poster identity and influence had significant and positive relationships with the number of likes and reposts, which is consistent with our hypothesis. In conclusion, these results suggest that it is feasible to explore the factors influencing the number of likes and reposts in the online mental community using the information adoption model, and the findings have important implications for users of online mental health communities as well as service providers.

The contributions of this study can be categorized into two main areas:

1. Theoretical contribution:

By cleverly combining the methods of text analysis and empirical analysis, we constructed a model of factors influencing the number of likes and reposts in online mental health communities that a post receives, based on the information adoption model. This study explores three particular variables of online mental health communities—the social experience topic, emotional expression topic, and the sentiment strength of posts by the users with depression from the perspective of information quality, considering the uniqueness of mental disorders. Not only does it construct a model of the factors influencing the number of likes and reposts in the online mental health communities, but it also confirms the applicability of the information adoption model on online mental communities.

2. Practical contribution:
  - To managers: The question of how to enhance user activity in an online community and how to provide users with mental illness with greater interaction has been a particular focus of the online mental health community. This study builds a model of factors influencing the number of likes and reposts applicable to the online mental community to help service providers to understand the key factors that enhance community user interaction. By testing the features of posts that users care about most in the online mental health community, we can propose some policies and suggestions for enhancing user communication in online mental health communities. For example, in terms of information quality, the community can help users sort out the topics they want to express by categorizing topics or entering prompt keywords; in addition, the community can encourage users to post posts with distinctive emotions, detailed content, and pictures to help them improve the quality of their posts. In terms of information source credibility, first, the community can not only use a more obvious way to indicate the authenticity of the user’s identity, but also should enhance the authentication for ordinary users who have been using the community for a long time; second, because the number of followers affects the number of recipients, the number of user‘s followers will affect the number of likes and reposts, so if the user’s posts are reporting a more crisis situation (such as a dangerous behavior), the community should proactively expand the dissemination of posts.
  - To users: Understanding the factors influencing the number of likes and reposts in the online mental health community is beneficial for community users to enhance their online communication skills and effectiveness. Not only can it help users to communicate more and gain more support within the community, resulting in better emotional understanding and release, but it can also provide people with mental illnesses with social therapy and a degree of relief and comfort.

### 5.2. Limitations and Future Research

There are some limitations of the current study. First, in addition to patients with diagnosed mental illnesses, there are also patients with undiagnosed illnesses and healthy users in the mental health online community, so it is difficult to study the postings of patients diagnosed with depression within the community. Second, the extent to which online community interactions currently help people with mental illness is difficult to measure and is beyond the scope of this study. Third, as we were unable to find other suitable online mental health communities at present, a single source of data in a single language means our findings may not robust, which is also a limitation of our research.

Thus, we propose a few directions for future research that could be carried out on this basis. First, future research could learn from posts’ comments to evaluate the effectiveness of user-to-user interactions. Second, a cross-comparison of more countries could be considered in future studies. Third, a hierarchical analysis could be performed between the independent variables in this study in order to identify relationships between them.

## 6. Conclusions

This study contributes to the investigation of the factors influencing the number of likes and reposts of posts in online mental communities, not only to help online communities to enhance the activity and sense of community among users but to help users with mental disorders to access online support. By combining text analysis and empirical research, we found that the users of online mental health communities are more attentive to the topics of social experience and emotional expressions, while at the same they care about the non-linguistic information. This highlights the importance of helping community users to post on community-related topics, and in terms of both information quality and source credibility, we provides policies and suggestions for administrators to help users gain the communication and support they need.

## Figures and Tables

**Table 1:** The definitions and descriptions of the variables.

Variables			Description
Dependent Variable	Perceived Usefulness	Number of likes and reposts (NLR)	Count of likes and reposts
Independent Variable	Information Quality	The topic of social experience in posts (SE)	The probability that a post discusses social experiences
		The topic of emotional expression in posts (EE)	The probability that a post discusses emotional expression
		Emotional strength (ES)	Emotion score of posts
		Length of posts	Number of text words
		Images of posts	Whether or not it contains emoji or pictures
	Information Source Credibility	Poster’s identity	Whether or not the poster has an id certification from the platform
		Poster’s influence	Logarithm of the number of poster’s followers
Control Variable		Life of posts	Number of days between posting and collecting day
		Number of post’s comments	Count of comments

**Table 2:** Descriptive statistics of variables.

Variables	Obs	Mean	Std.	Min	Max
ST	47,307	0.396	0.390	0	0.998
ET	47,307	0.260	0.343	0	0.998
ES	47,307	−1.737	8.683	−98.520	96.288
Length	47,307	15.820	23.281	1	812
Image	47,307	0.158	0.365	0	1
Identity	47,307	0.059	0.236	0	1
Influence	47,307	1.701	0.915	0	6.645
Life	47,307	20.138	11.578	0	40
Comment	47,307	7.947	50.255	0	4535
NLR	47,307	3.446	47.996	0	8651

**Table 3:** Correlation coefficient table.

Variables	ST	ET	ES	Length	Image	Identity	Influence	Life	Comment
ST	1.000								
ET	−0.471	1.000							
ES	−0.022	0.242	1.000						
Length	0.086	−0.068	−0.086	1.000					
Image	−0.073	0.157	0.068	0.110	1.000				
Identity	−0.007	0.037	0.028	0.037	0.086	1.000			
Influence	−0.021	0.050	0.024	0.040	0.115	0.377	1.000		
Life	0.015	0.002	−0.002	0.012	−0.010	−0.019	−0.010	1.000	
Comment	−0.013	0.040	0.010	0.016	0.027	0.030	0.034	0.008	1.000

**Table 4:** Model validation and hypothesis testing.

Dimension	Variables	Coef.	Std. Err.	*z*	*p* > |*z*|	Significance ^1^
Information Quality	ST	0.368	0.019	19.54	0.000	***
	ET	0.353	0.022	16.04	0.000	***
	ES	0.002	0.001	2.40	0.016	*
	Length	0.007	0.003	22.28	0.000	***
	Image	0.417	0.017	24.78	0.000	***
Source Credibility	Identity	0.164	0.029	5.76	0.000	***
	Influence	0.209	0.007	30.23	0.000	***
Control Variables	Life	0.002	0.001	3.10	0.002	**
	Comment	0.032	0.000	76.64	0.000	***
	constant	−0.510	0.208	−24.52	0.000	***
Obs		47,307				
Prob > chi2		0.000				
R^2^		0.139				

^1^ Significant level of path coefficient: * *p* < 0.05, ** *p* < 0.01, *** *p* < 0.001.

## Data Availability

All relevant data sets in this study are described in the manuscript.
